# “Violence” in medicine: necessary and unnecessary, intentional and unintentional

**DOI:** 10.1186/s13010-018-0059-y

**Published:** 2018-06-11

**Authors:** Johanna Shapiro

**Affiliations:** Department of Family Medicine, UC Irvine School of Medicine, Rte 81, Bldg 200, Ste 835; 101 City Dr. South, Orange, CA 92651 USA

**Keywords:** Violence and medicine, Structural violence, Metaphorical violence, Wounded healer, Medical humanities

## Abstract

We are more used to thinking of medicine in relation to the ways that it alleviates the effects of violence. Yet an important thread in the academic literature acknowledges that medicine can also be responsible for perpetuating violence, albeit unintentionally, against the very individuals it intends to help. In this essay, I discuss definitions of violence, emphasizing the importance of understanding the term not only as a physical perpetration but as an act of power of one person over another. I next explore the paradox of a healing profession that is permeated with violence sometimes necessary, often unintentional, and almost always unrecognized. Identifying the construct of “physician arrogance” as contributory to violence, I go on to identify different manifestations of violence in a medical context, including violence to the body; structural violence; metaphoric violence; and the practice of speaking to or about patients (and others in the healthcare system in ways that minimize or disrespect their full humanity. I further suggest possible explanations for the origins of these kinds of violence in physicians, including the fear of suffering and death in relation to vicarious trauma and the consequent concept of “killing suffering”; as well as why patients might be willing to accept such violence directed toward them. I conclude with brief recommendations for attending to root causes of violence, both within societal and institutional structures, and within ourselves, offering the model of the wounded healer.

## Background

What constitutes violence? The World Health Organization defines violence as “the intentional use of physical force or power, threatened or actual, against oneself, another person, or against a group or community, which either results in or has a high likelihood of resulting in injury, death, psychological harm, maldevelopment, or deprivation.” [1]. This definition is broader than that of the Oxford English Dictionary (“Behaviour involving physical force intended to hurt, damage, or kill someone or something”)., Its advantage is that by including the phrase use of… “power” it acknowledges that violence can be not only a physical act but an act of power of one person over another. The definition goes on to state that “intentionality” refers to the intent to employ physical force or exert power, but does not speak to motive, which may even, according to the perception of the perpetrator, be regarded as benevolent. Thus, WHO argues that while the action exercised (whether involving force or more generally “power”) must be consciously chosen, regardless of intent, anything that is injurious to another is an act of violence. In this case harm is not necessarily the intention, but the byproduct of action. Thus violence can occur without conscious intent and is not necessarily confined to physical harm.

We can find several examples in the literature of this kind of violence absent a conscious intention to harm. The nursing literature examines the concept of vertical violence, defined as “any act of violence, such as yelling, snide comments, withholding pertinent information, and rude, ignoring, and humiliating behaviors, which occur between two or more persons on different levels of the hierarchical system….” [2]. For decades, this same literature has also highlighted the problem of horizontal or lateral violence [3] in which “nurses covertly or overtly direct their dissatisfaction inward toward each other, toward themselves, and toward those less powerful than themselves.” [4].

Importantly, in both vertical and horizontal violence, such behaviors are not necessarily viewed punitively by their perpetrators, but rather as a rite of passage that builds “resilience” in young nurses [5]. In other cases, “the perpetrator of violence may be unaware that his/her actions are perceived adversely.” [2]. While lateral violence is usually attributed to the oppressed status of a particular group (such as nurses in the historically hierarchical structure of the healthcare system), it has also been argued that such violence is in part the result of the nursing profession’s preponderance of “walking wounded,” [6] individuals who have suffered secondary trauma as a result of their highly stressful work [7]. Increasingly, we have come to realize that, despite their privileged status in the healthcare hierarchy, physicians also suffer from secondary trauma and resultant burn-out, [8, 9], making them vulnerable to committing acts of vertical (medical student) or horizontal (collegial) violence, regardless of how unintended.

A related concept, again using the term “violence” and again stressing its unintended nature, is organizational violence. In this case, researchers have discovered that bureaucracy, including that of healthcare institutions, can have unintended negative consequences that are morally problematic. Organizational violence derives from Bourdieu’s theorizing of symbolic violence, defined as “…the kind of gentle, invisible, pervasive violence that is exercised through cognition and misrecognition, knowledge and sentiment, often with the unwitting consent or complicity of the dominated…. [and] embedded in the very modes of action and structures of cognition of individuals.” [10]. This definition, notable for the apparently paradoxical pairing of “gentle” and “violence,” emphasizes the role of such “violent” acts as a tool of social control.

More recently, the term symbolic violence has been refined to refer to the misuse or abuse of symbolic power, i.e., power that is used to dominate rather than emancipate. This parsing of symbolic violence stresses that the exercise of power in clinical situations is not in and of itself wrong, harmful, or “violent.” “The structural and symbolic power wielded by doctors is legitimate, socially conferred and indispensable for help and healing to occur.” [11]. Thus, organizational violence is a special case of the exercise of symbolic power in which “rather than being responsive to patients, professionals are increasingly required to respond to the imperatives of the evaluative bureaucracy.” [12].

Other uses of the term violence have been linked to gentrification [13], cultural appropriation [14], and various forms of speech [15]. It is possible to worry that casting such a wide net to identify forms of violence runs the risk of the word becoming irrelevant [16]. While legal definitions of terminology must be narrow and precise, in larger discourse there may be important purposes to expanding definitional terms. One reason for calling unintended harms a kind of “violence” is to overcome the relative ease with which such events are ignored, dismissed, or trivialized [16]. Employing the word “violence” is a conscious way of highlighting a continuum of violence that we would prefer to ignore. Certain harms not traditionally labeled as violence may produce similarly devastating effects as acts of physical violence. Given the implications of social control implicit in the theories of Boudrieu and others, it is reasonable to argue that calling attention to these issues in a bold way is justified as a reminder that significant harm to others, especially vulnerable others, can occur in many forms.

In the light of the above discussion, it can be illuminating to examine various forms of violence in medicine and speculate about the functions such violence serve.

### Acts of violence in medicine

It is a great irony that medicine, the epitome of a healing profession, is often filled with forms of violence, sometimes necessary, sometimes unintentional, almost always unrecognized or minimized. In these instances, the patient becomes a kind of victim, treated differently and damagingly by a physician who (usually unwittingly) has set aside the patient’s humanity. In one formulation, “Medical violence is a curious product of the physician’s arrogance, trappings of technique, and the laity’s faith that medicine can solve all problems” [17]. This early article acknowledges the sometimes dysfunctional dynamic at play between doctors and patients that can produce violent exchange. To more deeply understand violence in medicine, we must seek the roots of “physician arrogance;” why physicians utilize their “techniques” in certain ways; how the public can inadvertently collude with these processes out of its own largely unrecognized emotional and psychological needs; and how social and cultural capital inequalities make it difficult for patients to protest violent treatment.

Although little scholarly writing has been devoted to this topic, there are multiple examples of violence in medicine. From the dismembering of the human body that occurs in the first year of medical school during the anatomy course, to amputations, surgeries, and diagnostic or interventional procedures that cause pain, including the numerous uncomfortable and sometimes dangerous side effects of drugs intended to heal, there is a brutal dimension to medicine. Medicine often inflicts pain to avoid even greater pain or death; sometimes it succeeds in this goal and sometimes it does not. It could be argued that keeping people technically alive while prolonging a meaningless existence is in itself a type of violence. Most of these instances are clearly not designed to impose violence; indeed the intention is usually beneficent. Sometimes the violent act is necessary for the wellbeing of the patient; nevertheless, sometimes the violent act is unnecessary, and sometimes it results in harm.

Some of the more obvious examples of violence in medicine are violence to the body, represented in procedures and interventions that produce pain and sometimes long-lasting harm; structural violence, encompassing the systemic forces that especially disadvantage vulnerable and marginalized patients; metaphoric violence, the use of warlike, militaristic language in explaining disease and treatment; and the habit of speaking to or about patients (and others in the healthcare system, including medical students, nurses, and colleagues) in ways that minimize or disrespect their full humanity.

## Violence to the body

“Aegrescit medendo,” wrote Virgil. “The remedy is worse than the disease” [18]. Many patients have experienced this feeling as they endure assessment procedures and therapeutic interventions that produce transitory or chronic pain or dangerous, even life threatening side effects. Physicians obviously do not take pleasure in causing this pain. But because they must, their brains actually change in the way they regard suffering. Studies conclude that the brains of physicians react to viewing pain in others much less strongly than the brains of laypersons [19]. While this is in some way an adaptive response so that physicians are able to function effectively in the face of another’s suffering, it may also lead to a general tendency to dismiss, ignore, or trivialize patients’ negative experiences. This in turn may result in language and interactions that are insensitive at best, and confrontational, harsh, and intimidating at worst.

The cognitive dissonance that results from physicians simultaneously knowing that they are practitioners of a healing profession, yet must often impose considerable pain on their patients, can result in defensive coping strategies that minimize, sanitize, or fail to acknowledge the suffering that results, of course to their patients, but also to themselves. Consider the pediatrician who reassures her little patient, “The injection is only a pinch,” only to be met with disbelieving howls. More seriously, the oncologist who encourages a patient into yet another round of chemotherapy in the absence of therapeutic response may be doing more harm than good. The consequence is a fundamental dishonesty that can contribute to patient mistrust and despair; [20, 21] and to physician burn-out and cynicism [22].

It is worth asking whether intervention is always appropriate. Just because we can, should we? Is Virgil sometimes right? Over-diagnosis and overtreatment are growing concerns in medical practice [23]. If intervention is judged to be both necessary and beneficial, then should we look for a more honest balance in physician disclosures between unduly alarming the patient and preparing the patient for suffering? These issues obviously come into play in end of life scenarios, and present a telling example of how physicians’ conscious intention not to do harm by destroying hope can instead impose unintended violence on the patient [24].

## Structural violence

Structural violence refers to social structures that impede individuals, groups and societies from reaching their full potential [25]. In medicine, it means institutions and established societal modes of functioning that lead to impairment and limitations in human life [26]. Their existence is so normalized and established that they are almost invisible and therefore either willfully or naively overlooked or ignored [27]. Structural violence is based on the idea that certain societal patterns (of social relations and roles, economic arrangements, institutional practices, laws etc.) are so firmly entrenched that they are perceived as a “given,” just the way things are. Sources of suffering are deeply embedded in these ordinary, taken-for-granted patterns, including ill health and the inability to adequately access remedies.

The concept of structural violence encourages us to recognize that dimensions of life we might regard as disconnected are actually interrelated. The existence of structural violence makes it easier for privileged physicians to see themselves as distinct and separate from their patients, rather than implicated in the institutions they serve and the jobs they benefit from. When illness is perceived through an exclusively biomedical, molecular lens, it is unlikely physicians will emphasize the social determinants of illness [24]. These limits in perspective often result in physicians seeing under-resourced patients as responsible for their own problems, not “caring” about their health, or not choosing health as their highest priority. Structural violence results in harm to patients both directly, through disadvantaging them in terms of health and access to healthcare; and indirectly, by allowing physicians to adopt simplistic patient-blaming attitudes that ignore the larger structural issues.

## Violence in language

We also find examples of linguistic violence in medicine, which take two forms: violent metaphors and punishing, bullying language.

### Violent metaphors

Metaphors are commonly employed by physicians in clinical care [28] and research suggests patients like physicians who use metaphor better than those who do not. So it is important to ask, how do metaphors in medicine and violence intersect? A recent issue of the *American Journal of Bioethics* [29] was devoted to a discussion of the prevalence of military, warlike metaphors in medicine, and what this might mean for the practice of medicine. For example, in the metaphor “illness is war,” illness is what linguists refer to as the target conceptual domain and war is the source conceptual domain [30]. This means that insights about illness will come from what we know about war, thus fundamentally influencing our understanding of illness in a violent, combative direction. Similarly, as Fuks [31] points out, military metaphors may give undue emphasis to physical, biological aspects while ignoring psychological, spiritual, social dimensions of illness and healing. This imbalance may have the effect of silencing patients’ voices about subjective experiences of illness [32]. It has been argued that the reliance on war metaphors in medicine in medicine may contribute to patient anxiety and fear [33] as well as over-diagnosis and overtreatment [34].

Violent metaphors in cancer have been criticized because they imply that succumbing to the illness is a defeat and a failure [35, 36]. Patients who view disease as an “enemy” have higher levels of depression and anxiety; patients encouraged to “fight” may feel they have to suppress their emotional distress and maintain a positive attitude to avoid upsetting family members and physicians [37]. A corpus analysis of physician writing and speech identifies the most prevalent violence metaphor as one of fighting and protecting [38]. Such metaphors put the physician in a heroic light, while the patient is reduced to a foot soldier, or worse, a battleground. More complex analyses, however, highlighting agency, point to the conclusion that physicians often employ violent metaphors as an acknowledgment of “institutional barriers to good care and …how current systems and practices do not always benefit patients” [39].

Bleakley et al. [40] offer some alternative metaphors, seeking to shift the way physicians think, the way they speak, and the way they behave by urging language of collaboration, exploration, and journey. These authors argue that collaboration metaphors may beneficially affect clinical practice by “turning attention away from an disembodied agent of illness that must be eradicated to an embodied person in need of care.” Nie et al. [32] also contribute the metaphor of a journey as one that emphasizes humanizing and personal growth dimensions of healing, while placing the patient rather than the physician at the center of the narrative.

Some scholars have made a case for the value of militaristic metaphors, observing that they can empower patients; [41] and might have special utility in emergent situations [42]. Perrault and O’Keefe [43] advocate for plural or mixed metaphors tailored to the needs of particular patients. These authors argue that metaphors are not inherently good or bad, but must be judged in the context of the particular patient and situation [44].

### Demeaning interactions

Demeaning interactions with patients or disparaging comments about patients to learners, colleagues, or other health professionals fit the WHO definition of “use of power” resulting in psychological harm and deprivation of dignity, respect, and humanity. Familiar examples of such linguistic rationalizations include: “Patient is noncompliant,” “Patient does not appear to care about her health.” “Patient is demanding, uncooperative, hostile etc.” Other language may be considered violent, angry, confrontational, or bullying: [40] “Patient failed the chemotherapeutic regimen.” “Patient is a poor historian.”

Bleakley et al. [40] emphasize the link between metaphor and performance. When our thought patterns and verbalizations are grounded in combative, warlike metaphors, it is more likely that these will influence our interactions and behavior. These linguistic forms of discourse may result in patients feeling blamed for their lack of success in regaining health or intimidated by the physician [45]. This is a permutation of violence that demeans and belittles the other. Other targets of such bullying or intimidating interactions are medical students, [46] nurses, staff, and even colleagues [47].

## Origins of violence in medicine

In this section, I try to look beneath the surface of the decision-making, actions, language, and even structures in medicine that can be conceptualized as violent or as having violent implications for patients, or certain categories of patients. These thoughts are not meant to replace sophisticated and complex analyses of structural violence or linguistic usage rooted in webs of economic and societal privilege; rather they are intended merely to add a further dimension for consideration and investigation. While I consider physicians and patients separately, my ultimate contention is that both groups share a common underlying fear of suffering and death related to the trauma of serious medical illness. This existential fear may predispose physicians to violent actions and patients to tolerance of such actions.

### The role of vicarious trauma

Vicarious traumatization is a term that describes the undesirable outcomes of working directly with traumatized populations, including negative interactions with patients and colleagues, and deleterious personal consequences [48, 49]. Although the literature tends to refer to specific types of traumatized patients, such as victims of physical/sexual abuse or natural/human-caused disasters, I maintain that simply by their very nature, suffering, pain, and dying can be traumatic events for many patients, regardless of the specific context in which they occur. By extension, healthcare itself, especially when provided by overworked, pressured, stressed care-givers, may produce vicarious trauma. Such secondary traumatic stress results in failures of empathy [50] which ultimately prepare the way for the above forms of medically-related violence.

Although physicians are professionally intimate with suffering and death, this does not mean that they necessarily become able to compassionately witness suffering or have resolved the fundamental dilemma in medicine that death will always be the final outcome for every patient. Indeed, some scholars have asserted that physicians enter medicine because of a particular fear of death [51]. Thus a deep-seated psychological cause of violence in medicine paradoxically may be inextricably tangled with the original raison d’etre for medicine – i.e., the alleviation of suffering and the resistance to death. The fear of suffering and death, as well as repeated exposure to the inevitability of these phenomena and the limits of medicine to forestall them, may lead some physicians to attitudes of defense and denial. The result is often callousness or even brutality, motivated by a need to “kill” or vanquish suffering, but which can all too easily become confused with the patient who is enduring (and thus confronting the physician with) said suffering.

To “kill” suffering, it is understandable that one might have to think of oneself as fundamentally different and separate from ordinary and vulnerable patients. By keeping a firm boundary between the roles of physician and patient, the physician may unconsciously attempt to insulate him or herself from the trauma of suffering and death. This may have to do with a deep fear of contamination [52] by the very person the physician is consciously trying to aid. Keeping the “other” carefully demarcated, even while attempting to assist them, can confer a sense of safety for the physician; but can also produce an objectification and diminution of the humanity of the patient. This understandable desire to avoid suffering may also be relevant in physicians’ resistance to structural interpretations of health and illness. As has been noted, structural violence injures some, but protects and benefits others. Acknowledging that one is implicated in the suffering of others is a painful realization. To safeguard themselves, many physicians might prefer to avoid it.

Aggressive action in medicine is aimed at “killing” suffering and vanquishing death. Violent metaphors support waging this battle. Bullying language pushes away the weak, undoctor-like patient who has succumbed to disease and vulnerability, Structural violence reinforces and protects physician privilege. These are all mechanisms for defending against physician existential vulnerability emanating from the experience of vicarious trauma while at the same time affirming their control and power.

### Why patients sometimes tolerate medical violence

Physicians are not alone in their fear and subsequent denial of suffering and ultimately death. As a result of the inherent traumatic nature of experiencing illness, these anxieties are intensified in patients as well [53]. Although patients are the victims of violence in medicine, nevertheless their persistent beliefs about the power of medicine to eliminate suffering and postpone death may have the unintended consequence of allowing or enabling this violence. As an intrinsically vulnerable population, patients are susceptible to multiple forms of violence. Faced with the ordeal of ongoing distress and perhaps impending death, the willingness to make a trade-off between acceptance of a certain amount of violence for the potential reduction of such misery becomes understandable, even appealing.

Despite the fact that the god-like status of physicians so prominent in the second half of the twentieth century has become considerably qualified through concepts such as patient-centered medicine [54] and shared decision-making, [55] in extremis many patients and families want to believe that the physician is in control and can accomplish miracles that are beyond mere mortals. Thus they still sometimes reflexively enjoin physicians to do “everything possible” [56]. This understandable desire for life at times may result in problematic and ultimately futile treatments. It may also encourage the use of warlike metaphors on the part of patients and families, as well as physicians. It may even indirectly intimidate patients and families into accepting “violent” (in the sense of dehumanizing) interactions because of the need to perceive physicians as all-powerful and all-knowing. Thus patients, families, and physicians may collude in acts of unintentional violence with the goal of eliminating suffering and preserving life.

Further, the unequal distribution of power, entrenched throughout history in the relationship between physicians and their patients and exacerbated by issues of race, gender, and class, reinforces this tendency to tolerate rather than question medical violence. Social inequalities and differences in cultural health capital [57] disadvantage many patients. Patients and their families often feel at the mercy of their doctors and medical teams. Protesting a racially biased remark or a rough handling may pale in comparison to the perception that their life or the life of their loved one hangs in the balance.

## Conclusion

The writer Daniel Jose Older argues that “the central act of violence is erasure.” The forms of violence described above often result in the erasure of the autonomy and dignity of those targeted intentionally or otherwise. Patients, family members, trainees and colleagues suffer the traumatic consequences of their illnesses and their caregiving, only to be burdened further by the thoughtless and insensitive exercise of power. What can be done to ameliorate these harmful outcomes?

I have noted that violence in medicine can comprise necessary and intentional acts consciously designed to increase patient well-being as well as unnecessary and accidental consequences of structural, linguistic, and behavioral physician practices.. Both are problematic and require modification in terms of how we manage them. Regarding the former, not all violence in medicine can be avoided – physicians work with the tools they have. But there is something fundamentally dishonest about the way this sort of violence is not acknowledged by physicians. It is a kind of foundational lie that contaminates much of doctor-patient interactions. If physicians were better able to honestly witness suffering, and own the pain they must at times cause, they might be better able to speak more openly about necessary pain and risk as well as hoped-for outcomes. This point is especially relevant to the whole notion of informed consent and medical decision-making, whose purpose is to provide patients with a realistic and accurate understanding of risks and benefits, yet is sometimes honored more in the breach than in the observance [58]. Even as medicine works hard to minimize suffering, perhaps it also needs to accept when suffering is unavoidable, and help patients address this suffering with the strength of compassion rather than of violence.

Regarding the latter category of inadvertent harm, once we recognize that the inevitable violence of medicine should not be compounded by unnecessary violence, we should encourage physicians to seek out alternative ways of speaking, interacting, and being. We can work to change the acts, structures, and language that physicians currently often rely on. For example, we can consider the costs and benefits of medical acts within the context of patients’ values and lives. We can work to dismantle societal and institutional structures that benefit some while significantly harming others and change the way we educate future physicians to recognize how larger social forces negatively impinge on the possibilities of the health and wellbeing of so many [25]. We can change our metaphors and reject as unacceptable and unprofessional language that demeans or insults patients, or that bullies and intimidates subordinates. All of this involves changing the culture of medicine.

Ultimately, however, in order to effectively change actions, structures, and language, we must also reconsider underlying attitudes that contribute to the persistent endurance of these patterns. One place to start is to help physicians develop an awareness of the capacity for suffering they *share* with their patients; even as they help patients accept the limits of medicine. This means blurring some of the boundaries between physicians and patients, recognizing their common humanity and therefore their collective vulnerability, fragility, and woundedness. It means helping patients realize that doctors are always their witnesses, guides, advisors, partners and consolers, but not always their saviors. It means helping physicians come to terms with the suffering and death they have witnessed so that they can help contain their patients’ anxiety and dread, while at the same time honestly acknowledging the pain they must sometimes inflict and sometimes feel. Recognizing solidarity, as well as difference, with patients might also predispose physicians to being more receptive to the insights of structural analyses and working toward changing social systems in recognition of the injustices they impose on patients.

Drawing on the nursing literature, medicine might benefit from incorporating the theory of the wounded healer into training and practice. In this formulation, physicians, like nurses, must acknowledge both personal and professional traumas that over the years have turned them into “walking wounded” who attempt to cope with their own pain by depersonalizing and/or lashing out (often unconsciously) at others. Those who have theorized this concept offer the hope that, while perfection is not possible, healing can arise from the very woundedness of the healthcare provider [7]. Conti-O’Hare has posited a mechanism for transforming personal and professional pain into growth and development, thus moving healthcare providers from “walking wounded” to wounded healer [59]. Komisar and McFarland note that when resident-physicians are able to find meaning in traumatic patient care situations, it contributes to a positive transformation of their vicarious trauma [50].

When physicians are unable to honestly confront and acknowledge suffering; when out of fear they deny their privilege and the way in which healthcare systems often disenfranchise the patients they are trying to serve; when they inappropriately indulge in violent language out of self-protection and a desire to establish a heroic, invincible image – all these result in harm to patients, families, staff, and colleagues. Acknowledging the multifactorial nature of instances of structural, linguistic, and behavioral acts of violence in healthcare and then intervening to address societal and individual root causes represent first steps in mitigating their deleterious effects. Healing woundedness by cultivating attitudes of compassion, solidarity, humility, and social justice may provide the foundation for an alternative vision of medical practice.
